# Changing expression profiles of lncRNAs, mRNAs, circRNAs and miRNAs during osteoclastogenesis

**DOI:** 10.1038/srep21499

**Published:** 2016-02-09

**Authors:** Ce Dou, Zhen Cao, Bo Yang, Ning Ding, Tianyong Hou, Fei Luo, Fei Kang, Jianmei Li, Xiaochao Yang, Hong Jiang, Junyu Xiang, Hongyu Quan, Jianzhong Xu, Shiwu Dong

**Affiliations:** 1 Department of Orthopedics, Southwest Hospital, Third Military Medical University, Chongqing, China; 2 Department of Biomedical Materials Science, School of Biomedical Engineering, Third Military Medical University, Chongqing, China; 3 Department of Anatomy, Third Military Medical University, Chongqing, China; 4 China Orthopedic Regenerative Medicine Group (CORMed), Chongqing, China

## Abstract

Bone is a dynamic organ continuously undergoing shaping, repairing and remodeling. The homeostasis of bone is maintained by the balance between osteoblastic bone formation and osteoclastic bone resorption. Osteoclasts (OCs) are specialized multinucleated cells derived from hematopoietic stem cells (HSCs) or monocytes/macrophage progenitor cells. There are different stages during osteoclastogenesis, and one of the most important steps to form functional osteoclasts is realized by cell-cell fusion. In our study, microarray was performed to detect the expression profiles of lncRNA, mRNA, circRNA and miRNA at different stages during osteoclastogenesis of RAW264.7 cells. Often changed RNAs were selected and clustered among the four groups with Venn analysis. The results revealed that expressions of 518 lncRNAs, 207 mRNAs, 24 circRNAs and 37 miRNAs were often altered at each stage during OC differentiation. Gene ontology (GO) and Kyoto Encyclopedia of Genes and Genomes (KEGG) biological pathway analysis were performed to predict the functions of differentially expressed lncRNAs and co-expressed potential targeting genes. Co-expression networks of lncRNA-mRNA and circRNA-miRNA were constructed based on the correlation analysis between the differentially expressed RNAs. The present study provided a systematic perspective on the potential function of non-coding RNAs (ncRNAs) during osteoclastogenesis.

Osteoclasts (OCs) are bone-specific multinuleated cells derived from hematopoietic stem cells (HSCs) or monocytes/macrophage progenitor cells responsible for bone resorption^1^. In physiological conditions, osteoclasts are crucial in maintaining bone homeostasis and dynamic remodeling. On the other hand, dysregulation of osteoclasts is one of the main characteristics of bone disorders such as osteoporosis, osteopetrosis and rheumatoid arthritis (RA)^2^. Two of the most important regulating factors during osteoclasts differentiation are receptor activator of nuclear factor κB ligand (RANKL) and macrophage-colony stimulating factor (M-CSF)^3^. Apart from these two main regulating factors, osteoclasts can also be activated by cytokines such as interleukin-17 (IL-17), interferon-γ (IFN-γ) and tumor necrosis factor-α (TNF-α)^4^,^5^,^6^. Mononuclear OCs start to express specific markers including tartrate-resistant acid phosphatase (TRAP), integrin α_v_β_3_, and matrix metalloproteinase 9 (MMP9)^7^. Although mononuclear OCs can also resorb bone, multinucleation brought about by cell-cell fusion is the most characteristic feature of mature osteoclasts. Mature OCs are highly polarized cells with new cytoskeletal structures such as a sealing zone and ruffle borders for more efficient bone resorption activity^8^. During the past decades, there has been an increasing interest in discovering novel regulating factors on osteoclastogenesis. Efforts have been made on studying the role of miRNAs, natural compounds, epigenetic regulations, etc^9^,^10^,^11^. However, more is to be explored based on the recent advances in biology.

Long non-coding RNAs (lncRNAs) are ancient yet newly recognized regulating molecules that have come into scene recently. The length of lncRNAs transcripts range from 200 nt to 100 kb. The expression profile of lncRNAs is tissue-specific and alters across various stages of cell differentiation. In recent years, a certain amount of studies were performed exploring the expression profile of lncRNAs in different cell types and diseases that enriched the raw data in studying its underlying functions^12^,^13^. Although several lncRNAs have been proved critical in regulating cellular processes and diseases, most of the functions of these lncRNAs remain unclear. The biological function of lncRNAs is multi-faceted, which vary differently from its locations, binding sites and acting modes^14^. The regulating role of lncRNAs is not solitary but through a large complex network that involves mRNAs, miRNAs and proteins^15^. In this consideration, we performed a systematic study in understanding the function of lncRNAs together with mRNAs.

Circular RNAs (circRNAs) are another group of non-coding RNAs that are widely spread in animal cells. It was first identified in 1991^16^ and thought to be functionless as by-products in the following two decades. The biogenesis and potential function of circRNAs remains poorly understood, only two circRNAs (*ciRs-7/CDR1* and *Sry*) have been reported functional as microRNAs (miRNAs) sponges^17^,^18^. Similar with lncRNAs, studies showed that circRNAs expression profile is specific among different cell types indicating its possible regulatory function^19^. Recently, progresses have been made on the formation and biogenesis of circRNAs adding more evidence and possibilities of its biological value^20^,^21^. Apart from its potential regulating role in cellular processes, studies have been performed probing the promising role of circRNAs as biomarkers of diseases such as Alzheimer’s disease (AD) and tumor^22^,^23^.

To date, little is known about the functions of lncRNAs and circRNAs in skeletal system. Specifically, no report was made on the expression profiles of lncRNAs and circRNAs during osteoclastogenesis. In our study, we specifically focused on the cellular process of fusion partially because of its importance in forming mature osteoclasts; hopefully, the general occurrence of fusion under physiological or pathological conditions might give our data wider relevance.

Here we performed microarray analysis on the expression profiles of lncRNAs, mRNAs, circRNAs and miRNAs during osteoclasts differentiation and fusion at different stages using RAW264.7 cells. Gene ontology (GO) and Kyoto Encyclopedia of Genes and Genomes (KEGG) pathway analysis were done based on the function of mRNAs that change their expression levels over time in a positive or negative correlation with the changing lncRNAs. Co-expression and network potential targeting relationship were constructed according to the microarray results and bioinformatics predictions.

## Results

### Osteoclasts differentiation and specific genes expression change at different stages

**Figure 1:**
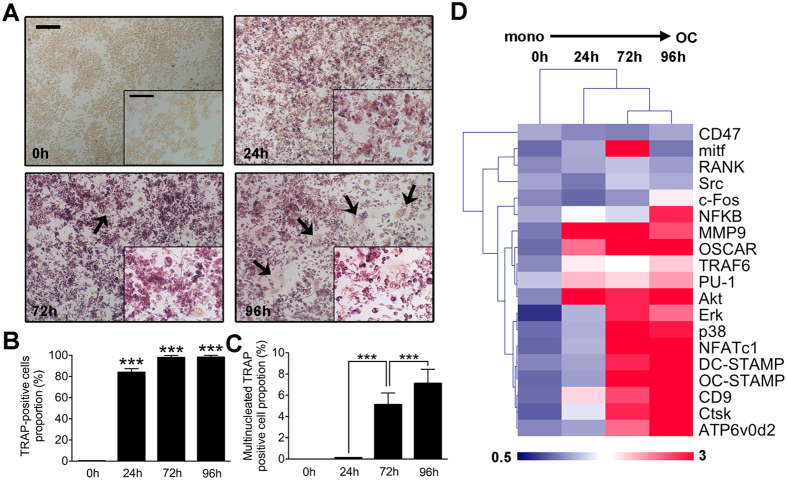
Osteoclasts differentiation and specific genes expression change at different stages. (**A**) Representative images of TRAP stain of osteoclasts cultured from RAW264.7 cells induced with RANKL (100 ng/mL) and M-CSF (50 ng/mL) for 0 h, 24 h, 72 h and 96 h. The upper scale bar represents 200 μm and the lower scale bar represents 50 μm. Multinucleated cells were highlighted by black arrows. (**B**) Quantitative analysis of TRAP positive cell proportion of at each stage during the differentiation. (**C**) Proportion of TRAP positive cells with more than three nuclei (three included) in total TRAP positive cells at each differentiation stage. (**D**) Osteoclasts specific and fusogenic genes expression level alterations from 0 h (monocytes) to 96 h (mature osteoclasts).The data in the figures represent the averages ± SD. *p < 0.05, **p < 0.01, and ***p < 0.001 based on one-way ANOVA.

In this study, we used RAW264.7 cells instead of primary bone marrow macrophages as osteoclast precursors. Not all the observed changes may be relevant for osteoclastogenesis *in vivo*. Cells were induced with RANKL (100 ng/mL) and M-CSF (50 ng/mL) for 24 h, 72 h and 96 h respectively. TRAP stain was performed to evaluate the differentiation of OCs (Fig. 1A). Quantitative analysis showed that TRAP negative cells at 24 h accounted for about 20 percent of total cells. At 72 h and 96 h, nearly all the cells became TRAP positive (Fig. 1B). Further analysis of TRAP-positive cells with more than three nuclei (three nuclei included) showed that cells induced for 96 h had most mature osteoclasts. In comparison, TRAP-positive cells with more than three nuclei (highlighted by arrows) were lesser in the other three groups indicating an increased fusion process between 72 h and 96 h (Fig. 1C). The gene microarray analysis of osteoclasts differentiation at different stages is consistent with the TRAP stain results showed that most RANKL-dependent gene expression was up regulated during osteoclastogenesis (Fig. 1D). Specifically, fusion related genes such as *DC-STAMP* and *ATP6v0d2* were highly expressed at 72 h, and 96 h. In addition, two negative regulatory genes were both down regulated during OCgenesis in our *in vitro* model (Fig. S1).

### Osteoclasts formation and fusion at different stages

**Figure 2:**
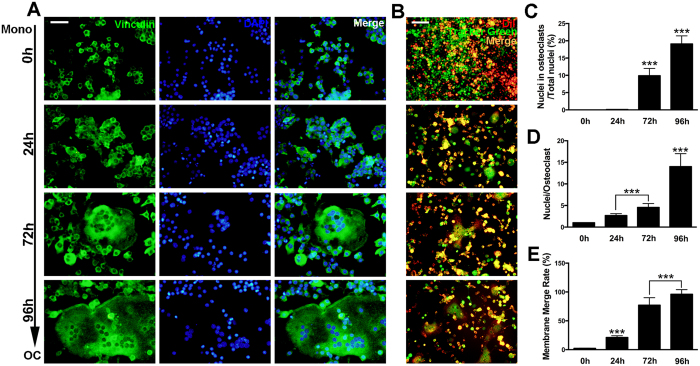
Osteoclasts formation and fusion at different stages. (**A**) Representative images of FAK stain of osteoclasts cultured from RAW264.7 cells induced with RANKL (100 ng/mL) and M-CSF (50 ng/mL) for 0 h, 24 h, 72 h and 96 h. The scale bar represents 100 μm. (**B**) Representative images of fusion assay. Cells were induced with RANKL (100 ng/mL) and M-CSF (50 ng/mL) for 0 h, 24 h, 72 h and 96 h. The scale bar represents 100 μm. (**C**) Quantification of the total number of nuclei in osteoclasts over the total number of nuclei. (**D**) Average nuclei number of osteoclasts of FAK stain at different stages. (**E**) Membrane merge rate of osteoclasts from fusion assay at each differentiation stage. The data in the figures represent the averages ± SD. *p < 0.05, **p < 0.01, and ***p < 0.001 based on one-way ANOVA.

Since cell-cell fusion is one of the most important processes during multinucleated OCs formation, we then performed actin cytoskeleton and focal adhesion (FAK) staining to further observe the cytoskeleton of osteoclasts at different stages (Fig. 2A). In accordance with the previous results, nuclei belong to osteoclasts over total nuclei increased significantly at 72 h and 96 h (Fig. 2C). As for average nuclei number, osteoclasts induced for 96 h showed the highest average nuclei number (Fig. 2D). In addition, fusion assays were performed to evaluate the membrane merge rate (Fig. 2B). The results showed that osteoclasts groups at 72 h and 96 h had significantly higher membrane merge rate (Fig. 2E). Particularly, the merging rate reached to almost 100 percent in the 96 h group.

### Osteoclasts sealing zone formation and bone resorption activity at different stages

**Figure 3:**
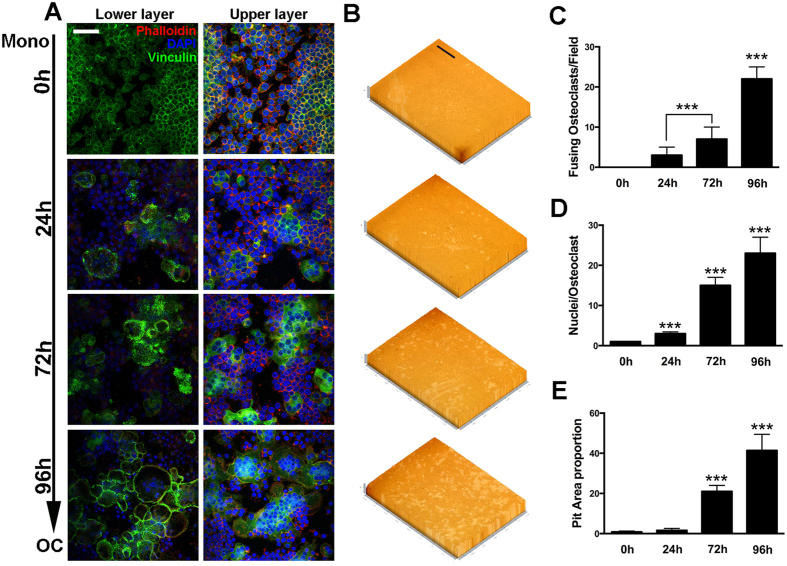
Osteoclasts sealing zone formation and bone resorption activity at different stages. (**A**) Representative images of FAK stain of osteoclasts at different stages during differentiation. Sealing zone was observed at lower layers with confocal microscopy. The scale bar represents 20 μm. (**B**) Representative images of pit formation assay. Cells were induced with RANKL (100 ng/mL) and M-CSF (50 ng/mL)for 0 h, 24 h, 72 h and 96 h.The scale bar represents 800 μm. (**C**) Number of fusing osteoclasts at different stages. (**D**) Average nuclei number of osteoclasts at different stages in FAK stain. (**E**) Quantification of pit area formation in (**B**). The data in the figures represent the averages ± SD. *p < 0.05, **p < 0.01, and ***p < 0.001 based on one-way ANOVA.

Increased osteoclasts size due to cell-cell fusion is usually associated with increased osteoclastic function. To further evaluate the function of osteoclasts at different stages, confocal microscopy was used to observe the formation of sealing zone (Fig. 3A). Cells were observed on different layers at the same position for a more comprehensive view. As shown in the results, with the appearance of large multinucleated osteoclasts, the formation of sealing zones increased in consistency. Quantitative analysis showed that fusing osteoclasts number is significantly higher in the 96 h groups although it seemed to be a massive large cell from the upper layer (Fig. 3C). The extensive formation of sealing zone and fusion process implied an increased osteoclastic activity. Pit formation was then performed for verification (Fig. 3B). As expected, osteolcasts in the 96 h group showed significantly higher bone resorption activity (Fig. 3E). In addition, pit formation was also verified by scanning electron microscope (SEM) analysis (Fig. S2).

### Expression profiles of lncRNAs and mRNAs during osteoclastogenesis

**Figure 4:**
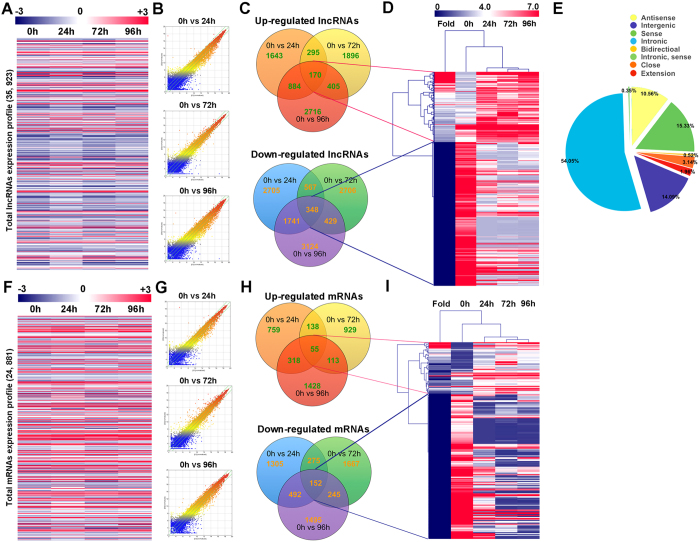
Expression profiles of lncRNAs and mRNAs during osteoclasts differentiation and fusion. (**A**) The cluster heat map of all lncRNAs expression at different stages during osteoclastogenesis from microarray data. (**B**) Scatter plots showing differentially expressed lncRNAs between osteoclasts at different stages and monocytes (0 h). (**C**) Often differentially expressed lncRNAs between osteoclasts at different stages and monocytes (0 h). (**D**) Hierarchical clustering showing often up and down regulated lncRNAs among the four groups. (**E**) lncRNAs categories and distribution in the microarray. (**F**) The cluster heat map of all mRNAs expression at different stages during osteoclastogenesis from microarray data. (**G**) Scatter plots showing differentially expressed mRNAs between osteoclasts at different stages and monocytes (0 h). (**H**) Often differentially expressed mRNAs between osteoclasts at different stages and monocytes (0 h). (**I**) Hierarchical clustering showing often up and down regulated mRNAs among the four groups.

Total RNA was extracted from osteoclasts at different stages as previously described. Arraystar Mouse lncRNA microarray V3.0 was adopted for the profiling of mouse lncRNAs and protein-coding transcripts. In total, 35,923 lncRNAs were detected and the whole expression profile was presented (Fig. 4A), 24,881 mRNAs were detected and the whole expression profile was presented (Fig. 4F). Three comparison groups were set according to the differentiation stages of osteoclasts, 0 h *vs* 24 h (pre-osteoclasts), 0 h *vs* 72 h (mature osteoclasts), and 0 h *vs* 96 h (activated osteoclasts) (Fig. 4B,G). For pre-osteoclasts, 1,643 lncRNAs and 759 mRNAs were up regulated, 2,705 lncRNAs and 1,305 mRNAs were down regulated (Fig. 4C,H). For mature osteoclasts, 1,896 lncRNAs and 929 mRNAs were up regulated, 2,706 lncRNAs and 1,667 mRNAs were down regulated (Fig. 4C,H). For activated osteoclasts, 2,716 lncRNAs and 1428 mRNAs were up regulated, 3,124 lncRNAs and 1,495 mRNAs were down regulated (Fig. 4C,H). All the differentially expressed lncRNAs and mRNAs were statistically significant (*p* < 0.05) with fold change greater than 2.0. VENN analysis revealed that 170 lncRNAs and 55 mRNAs were often up regulated and 348 lncRNAs and 152 mRNAs were often down regulated at all the stages during OCgenesis (Fig. 4C,H). A cluster was generated and analyzed with hierarchical clustering (HCL) for the often differentially regulated 170 lncRNAs (up) and 348 lncRNAs (down) (Fig. 4D). In the same way, a cluster was generated and analyzed with HCL for the often differentially regulated 55 mRNAs (up) and 152 mRNAs (down) (Fig. 4I). Subgroup analysis showed genomic classification and distribution of all lncRNAs (Fig. 4E). The results suggested that among differentially expressed lncRNAs, intronic lncRNAs occupied 54.05% followed by 15.33% of sense lncRNAs and 14.09% intergenic lncRNAs. The results were similar with the lncRNAs expression profiles in other models^24^,^25^,^26^, suggesting that lncRNAs expression pattern is fixed through various tissues and systems. The expression of top 10 often upregulated and downregulated lncRNAs were validated by qPCR (Fig. S3A–J). We also extracted and clustered the differentially expressed lncRNAs and mRNAs (fold change >2.0, *p* < 0.05) in all comparison groups for better observation of expression patterns (Fig. S4).

### Expression profiles of circRNAs and miRNAs during osteoclastogenesis

**Figure 5:**
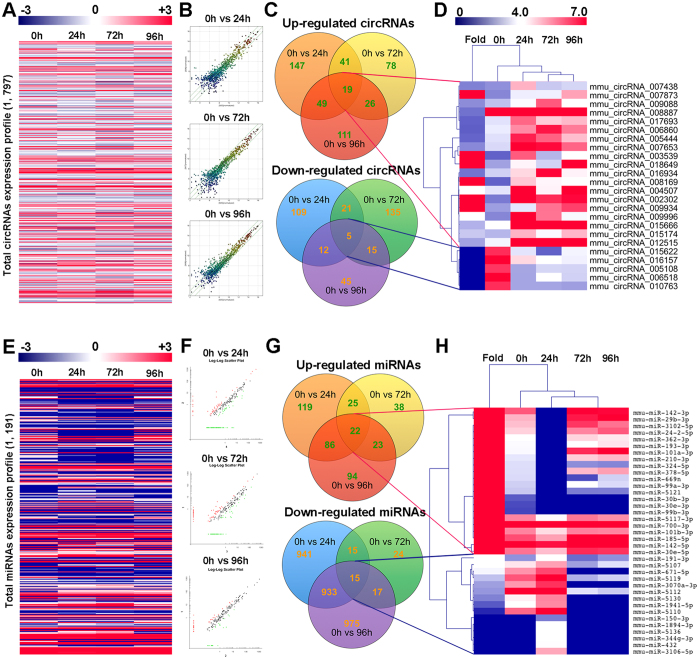
Expression profiles of circRNAs and miRNAs during osteoclasts differentiation and fusion. (**A**) The cluster heat map of all circRNAs expression at different stages during osteoclastogenesis from microarray data. (**B**) Scatter plots showing differentially expressed circRNAs between osteoclasts at different stages and monocytes (0 h). (**C**) Often differentially expressed circRNAs between osteoclasts at different stages and monocytes (0 h). (**D**) Hierarchical clustering showing often up and down regulated circRNAs among the four groups. (**E**) The cluster heat map of all miRNAs expression at different stages during osteoclastogenesis from microarray data. (**F**) Scatter plots showing differentially expressed miRNAs between osteoclasts at different stages and monocytes (0 h). (**G**) Often differentially expressed miRNAs between osteoclasts at different stages and monocytes (0 h). (**H**) Hierarchical clustering showing often up and down regulated miRNAs among the four groups.

Total RNA of osteoclasts at different stages was extracted. Arraystar Mouse circRNA Array analysis was adopted for profiling the mouse circRNAs expression (Fig. 5A). Agilent miRNA microarray was adopted for profiling of the mouse miRNAs expression (Fig. 5E). In total, 1,797 circRNAs were detected and the whole expression profile was presented (Fig. 5A), 1,191 miRNAs were detected and the whole expression profile was presented (Fig. 5E). Three comparison groups were set according to the differentiation stages of osteoclasts as described above (Fig. 5B,F). For pre-osteoclasts, 147 circRNAs and 119 miRNAs were up regulated, 109 circRNAs and 941 miRNAs were down regulated (Fig. 5C,G). For mature osteoclasts, 78 circRNAs and 38 miRNAs were up regulated, 135 circRNAs and 24miRNAs were down regulated (Fig. 5C,G). For activated osteoclasts, 111 circRNAs and 94miRNAs were up regulated, 45 circRNAs and 975miRNAs were down regulated (Fig. 5C,G). All the differentially expressed circRNAs and miRNAs were statistically significant (*p* < 0.05) with fold change greater than 2.0. VENN analysis revealed that 19 circRNAs and 22miRNAs were often up regulated and 5 circRNAs and 15miRNAs were often down regulated at all the stages during OCgenesis (Fig. 5C,G). A cluster was generated and analyzed with hierarchical clustering (HCL) for the often differentially regulated 5 circRNAs (up) and 22 circRNAs (down) (Fig. 5D). In the same way, a cluster was generated and analyzed with HCL for the often differentially regulated 22 miRNAs (up) and 15 miRNAs (down) (Fig. 5H). The expression of top 10 often upregulated and downregulated circRNAs were validated by qPCR (Fig. S3K–T). We also extracted and clustered the differentially expressed circRNAs and miRNAs (fold change >2.0, *p* < 0.05) in all comparison groups for better observation of expression patterns (Fig. S5).

### Construction of the lncRNA-mRNA co-expression network

**Figure 6:**
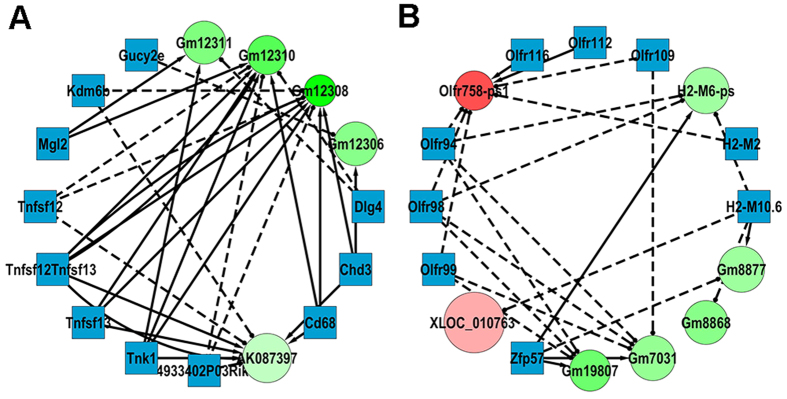
Construction of the lncRNA-mRNA co-expression network. Circle nodes represent lncRNA and square nodes represent mRNAs. Red color and green color represent up and down regulation respectively. The shade darkness of red and green represents fold change of lncRNAs. The size of circle represents p-value with larger size owing smaller p-value. Solid lines represent positive relationship and dash lines represent negative relationship. (**A,B**) Detailed presentation of two sub-networks in the dashed box in Fig. S6.

Based on the data analysis results, we constructed a lncRNA-mRNA co-expression network. 1,442 pairs of lncRNA and mRNA relationships (including repeated ones) were selected with significant values of Pearson correlation coefficients (*p* < 0.05). A total 241 lncRNAs and mRNAs containing 334 relationships were selected to generate a network map (Fig. S6). Two sub-networks containing 32 lncRNAs and mRNAs and 56 relationships with most lncRNA-mRNA interactions were presented in detail (Fig. 6). The network implied a complex regulating relationship between lncRNAs and mRNAs. One lncRNA could regulate multiple genes in different ways while one gene could be regulated by multiple lncRNAs. From the network we found that tumor necrosis factor ligand superfamily member (Tnfsf)12 and Tnfsf13 were intimately related with lncRNA Gm12310 and Gm12308 (Fig. 6A). It is well known that proteins encoded by *Tnfsf12* and *Tnfsf13* could induce apoptosis in a cell-type specific manner. However, both proteins were stimulative in osteoclastogenesis^27^,^28^. It is also interesting to notice that in Fig. 6B, mRNAs of olfactory receptor (Olfr) 94/98/99/109/112/116 were all correlated with lncRNA Olfr758-ps1, but no report was made describing the function of these olfactory receptors in osteoclastogenesis.

### GO analysis of the biological function of lncRNA co-expression genes

**Figure 7:**
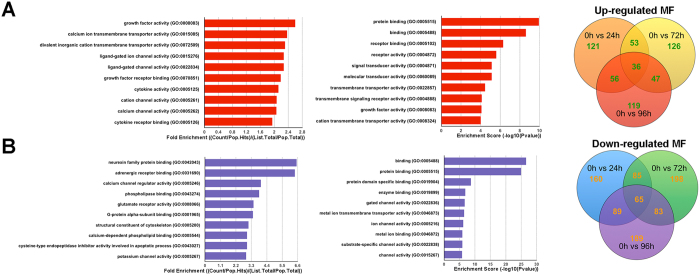
GO analysis of the biological function of lncRNA co-expression genes. (**A**) Often up regulated GO MF terms for the difference lncRNAs co-expression genes were analyzed. Top 10 often up regulated GO terms ranked by fold enrichment and enrichment score were shown. (**B**) Often down regulated GO MF terms for the difference lncRNAs co-expression genes were analyzed. Top 10 often down regulated GO terms ranked by fold enrichment and enrichment score were shown.

Differentially regulated lncRNAs and co-expression genes were further analyzed by DAVID bioinformatics resources 6.7 (http://david.abcc.ncifcrf.gov). GO analysis were made on three different aspects namely biological process (BP), cellular component (CC) and molecular function (MF) for up regulated and down regulated lncRNAs respectively (Fig. 7 and Fig. S7). Prediction terms with p-value less than 0.05 were selected and ranked by fold enrichment ((Count/Pop. Hits)/(List. Total/Pop. Total)) or enrichment score (−log10(p-value)). According to the results, 725, 807 and 830 BP terms, 69, 41 and 55 CC terms, 121, 126 and 119 MF terms were found up regulated (*p* < 0.05) in all comparison groups. In contrast, 864, 1,073 and 1,083 BP terms, 104,149 and 145 CC terms, 160, 198 and 189 MF terms were found down regulated (*p* < 0.05). Top 10 generally changed GO terms in all comparison groups classified by BP, CC, MF and ranked by fold enrichment or enrichment score were listed. The most enriched and meaningful BP terms were related to immune response, calcium transport and cellular signaling such as *‘regulation of interferon-gamma production* (GO:0032729)*’, ‘immune response-regulating cell surface receptor signaling pathway* (GO:0002768)*’, ‘negative regulation of B cell activation* (GO:0050869)*’, ‘calcium ion transmembrane transport* (GO:0070588)’ and *‘cell communication* (GO:0007154)’. The most enriched CC terms were mostly about cell membrane such as *‘cell surface* (GO: 0009986)*’, ‘membrane part* (GO: 0044425)*’, ‘intrinsic to membrane* (GO: 0031224)*’, ‘filopodium* (GO: 0030175)’ and *‘dendrite cytoplasm* (GO: 0032839)*’.* As for MF, we found that the most enriched terms were also closely related with calcium transportation, receptor binding and cytoskeleton were also referred to. Represented terms were *‘calcium channel activity* (GO: 0005262)*’, ‘calcium channel regulator activity* (GO: 0005246)*’, ‘receptor binding* (GO: 0005102)*’, ‘calcium-dependent phospholipid binding* (GO: 0005544)’ and *‘structural constituent of cytoskeleton* (GO: 0005200)’. Moreover, KEGG pathway analysis was made, pathways (*p* < 0.05) were selected and ranked by gene counts. Top 20 pathways were listed for up regulated and down regulated lncRNAs respectively (Tables S3 and S4).

### Construction of the circRNA-miRNA co-expression network

**Figure 8:**
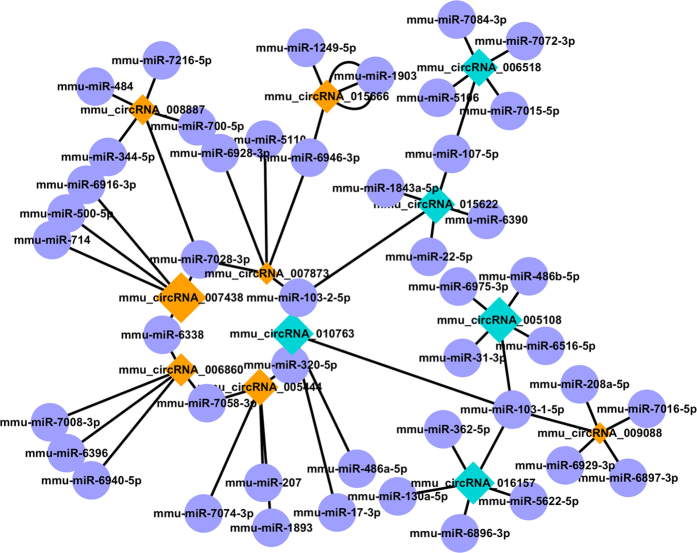
Construction of the circRNA-miRNA co-expression network. Construction of the circRNA-miRNA co-expression network. Diamond nodes represent circRNAs and purple circle nodes represent miRNAs. Yellow color and blue color represents up and down regulation respectively. The size of diamonds represents fold change of circRNAs with larger size owing higher fold change.

We then constructed a circRNA-miRNA co-expression network based on the microarray results. A network map was constructed containing 24 circRNAs, 82 miRNAs and 95 relationships (Fig. 8A). Sub-network that has the most interactions was presented in detail (Fig. 8B). In the network, circle represents miRNA and diamond represents circRNA. Yellow color and blue color represents up and down regulation respectively. The size of diamond represents fold change of circRNAs with larger size owing higher fold change. The network is simpler compare to that of the lncRNA-mRNA relationship. It is partially owing to the base number of detected circRNAs and miRNAs in the microarray is smaller. However, we could still figure out that there exists a core circRNA-miRNA regulation network during the process of osteoclastogenesis. In our results, all of the differentially expressed circRNAs in the circRNA-miRNA co-expression network were not annotated. However, some of the co-expressed miRNAs have already been proved functional in skeletal system. Among which miR-31 has recently been proved regulating osteoclastogenesis by targeting RhoA^29^. From the co-expression network we can see that miR-103 is co-related with one up regulated circRNA (circRNA_007873) and two down regulated circRNAs (circRNA_010763, circRNA_015622) at the same time.

The information regarding our data was submitted to the Gene Expression Omnibus, the accession number is GSE72478.

## Discussion

In our *in vitro* osteoclastogenesis system, RAW264.7 cells before 3 passages were treated with RANKL (100 ng/ml) and M-CSF (50 ng/ml). The first process from 0 h to 24 h represented the differentiation from monocytes to pre-OCs. Most cells became TRAP positive at 24 h (>80%). The second process from 24 h to 72 h represented differentiation from pre-OCs to mature OCs. Multinucleated (nuclei number >3) cells appeared (5%), membrane merge rate (76%), and bone resorption activity (21%) significantly increased. The third process from 72 h to 96 h represented activation of mature OCs. Activated OCs had most average nuclei number (about 13%), highest membrane merge rate (93%) and most efficient bone resorption activity (42%). Since RAW264.7 cells could not fully reflect osteoclast differentiation, further study using primary cells is in need. In our study, the expression profiles of lncRNA, mRNA, miRNA and circRNA were detected at these different stages. From the results, we figured out that at each stage during osteoclastogenesis, thousands of lncRNAs were differentially expressed compare to the control group. It is very interesting to notice that the expression pattern of lncRNAs was consistent with mRNAs that have more down regulated transcripts (Fig. 4). On the opposite, the expression patterns of circRNAs and miRNAs were with more up regulations and less down regulations (Fig. 5). This result collaboratively explained that most of the current studies focused on the relationship between lncRNA-mRNA pairs as well as circRNA-miRNA pairs. It is also worth mentioning that for both RNAs, expression pattern that are down at 24 h and 72 h but come back up at 92 h do not exist (Figs S4 and S5).

As a “bridge” between DNA and protein, the complex regulatory role of RNA has long been underestimated. In eukaryotic cells, protein-coding RNA (mRNA) only occupies about 2% of the genome, the rest massive number of transcripts were classified to non-protein coding RNAs (ncRNAs). Except for well-acknowledged ribosomal RNA (rRNA) and transfer RNA (tRNA), other short and long (>200 bp) ncRNAs were thought to be transcriptional “noises” once upon a time. However, accumulating evidences showed that ncRNAs play a critical role in cellular functions^30^,^31^. Among all the ncRNAs, miRNAs (20–24 nt) were most intensively studied for the last decade. These small miRNAs bind to the complementary site on the 3′ untranslated region (UTR) of targeting mRNAs called miRNA binding elements (MREs) and block protein translation or modulate mRNA stability on a post-transcriptional level^32^. Unlike miRNAs, the function of lncRNAs is poorly understood. Due to its length (>200 bp), lncRNAs can fold into secondary or higher orders of structure making it more flexible in targeting proteins or gene sites^33^. Moreover, the complexity of lncRNAs is increased by differential splicing and alternative transcription initiation sites^14^. Among thousands of circRNAs, only two of them were recently uncovered with function as miRNA sponges^17^,^18^. Although the biogenesis of circRNAs is still not fully understood, its expression profiles were found tissue-specific making it more predictable of the potential biological functions^20^.

The study of ncRNAs in skeletal system is generally rare. Pioneering studies were performed on the expression profile of lncRNAs during chondrogenic differentiation and osteogenic differentiation^25^,^34^. Moreover, reports were made on the relationship between lncRNAs and bone disorders such as osteoarthritis, osteoporosis and tumor^35^,^36^,^37^. Currently, studies of ncRNA regulation in osteoclasts are limited to the field of miRNAs. This is the first report on the changing expression of lncRNA, mRNA, circRNA and miRNA in osteoclastogenesis. We aim to arouse the attention of ncRNAs regulation in studying osteoclastogenesis. GO analysis was performed to further annotate the biological functions of differentially expressed lncRNAs and their target genes. We noticed that a significant amount of GO terms were related with immune system. This phenomenon is very interesting considering the important role of osteoclasts in osteoimmunology. The progenitor cells of both osteoclasts and immune cells reside in bone marrows in which multiple cytokines and numerous immunomodulatory signals concurrently regulate bone metabolism and immune responses^38^,^39^,^40^. Increasing attentions are now being paid on the role of lncRNAs in the immune system^26^,^41^. However, no combination of lncRNAs and osteoimmunology has been made. In accordance with the results of GO analysis, KEGG pathway analysis also showed that pathways related with receptor interaction, immune response and calcium signaling were among the top regulated ones (Tables S3 and S4).

From the lncRNA-mRNA co-expression network, we found that TNFSF12, TNFSF13 and Mgl2 were co-expressed with multiple lncRNAs forming a complex network. This phenomenon is consistent with the GO & Pathway analysis indicating the importance of immune response related signaling in OC differentiation and fusion. In our study, most of the lncRNAs in the co-expression network were not annotated yet. It is very much worthy to perform further study in revealing the underlying mechanisms of these lncRNAs. According to the two recently identified circRNAs (*ciRs-7/CDR1* and *Sry*), circRNAs might act as competing endogenous RNAs (ceRNAs) regulating the function of miRNAs^17^,^18^. An estimated more than 25,000 different circRNAs exist in human cells^42^. It is predictable that a great amount of work will be done exploring the role of circRNAs in the near future. In our predicted circRNA-miRNA co-expression network, miR-103 was reported inhibitory on osteoblast proliferation with stimulated microgravity^43^,^44^. Another miR-17 has also been proved regulatory in osteoblastic differentiation and osteosarcoma^45^,^46^. miR-320 in the center was shown targeting fatty acid synthase in osteosarcoma and adipocytic differentiation from human mesenchymal stem cells (MSCs)^47^,^48^. None of these miRNAs have been reported functional during osteoclastogenesis.

In conclusion, the present study quantified the different stages during osteoclastogenesis. Then, the expression profiles of lncRNA, mRNA, circRNA and miRNA were detected by microarray at these different stages. GO and KEGG pathway analysis were made to annotate the potential functions of differentially expression lncRNAs. Co-expression networks were constructed for both lncRNA-mRNA and circRNA-miRNA. We aim to inspire the interests of researchers in studying the role ncRNAs in osteoclasts and osteoclasts related bone disorders.

## Methods and Materials

### Reagents

RAW264.7 cells were obtained from the American Type Culture Collection (Rockville, MD, USA). Recombinant mouse RANKL and recombinant mouse M-CSF were purchased from R&D Systems (Minneapolis, MN, USA). The Osteo Assay Surface for Bone Resorption was purchased from Corning (NY, USA). The TRAP stain kit was obtained from Sigma-Aldrich (NY, USA). The Actin Cytoskeleton and Focal Adhesion Staining Kit was purchased from Millipore (Darmstadt, Germany). Alpha minimal essential medium (α-MEM) and fetal bovine serum (FBS) were purchased from Gibco (Life Technologies, USA). Penicillin-streptomycin solution was obtained from Hyclone (Thermo Scientific, USA). Membrane dye DiI and Cell Tracker Green were obtained from Life Technologies.

### TRAP staining

RAW264.7 cells were cultured in α-minimal essential medium (MEM) containing 10% FBS and 1% Penicillin-streptomycin solution with M-CSF (50 ng/ml) and RANKL (100 ng/ml). For TRAP stain, cells were cultured in a 96-well plate at a density of 5 × 10^3^ cells/well. Cells were fixed in 4% paraformaldehyde for 20 min and then stained with TRAP staining solution (0.1 mg/ml of naphthol AS-MX phosphate, 0.3 mg/ml of Fast Red Violet LB stain) according to the manufactrurer’s instruction. TRAP-positive cell number and multinucleated cell (>3 nuclei) were counted.

### Actin Cytoskeleton and Focal Adhesion Staining

Cells were incubated in 96-well plate (5 × 10^3^ cells/well) and induced with M-CSF (50 ng/ml) and RANKL (100 ng/ml). Procedures were described in previous study^10^. In brief, cells were washed and fixed for permeabilization. After blocking, primary antibody (Anti-Vinculin) was then diluted to a working concentration (1:300) in blocking solution, and cells were incubated for 1 hour at room temperature. Secondary antibody (Alexa Fluor 488 Goat Anti-Mouse IgG (H + L) Antibody, Invitrogen) ((1: 500) and TRITC conjugated Phalloidin (1: 500) was diluted in 1 × PBS and cells were incubated for 1 h at room temperature. Nuclei counterstaining was performed by DAPI (1: 1000) for 5 minutes followed by fluorescence microscopy and confocal microscopy observation.

### Fusion Assay

Fussion assay was adopted as described in previous study^49^. Cells were induced with RANKL (100 ng/ml) and M-CSF (50 ng/ml) in 6-well plates. Then, cells were labeled with either membrane dye DiI or cell content marker Cell tracker green. After incubation for 30 min at room temperature, cells labeled with DiI were scraped and put onto the well containing cells labeled with cell tracker green. The co-plated cells were then incubated together for 2 h before removal of the medium. Fluorescence microscopy was adopted for observation. Image J software was adopted for the analysis of membrane merge rate.

### Pit Formation Assay

Cells were incubated in 96-well plates (Corning Osteo Assay Surface), 2 × 10^3^ cells/well. Primary BMMs were incubated in 48-well plates covered with bovine bone slices, 1 × 10^4^ cells/well. Cells were induced with RANKL (100 ng/ml) and M-CSF (50 ng/ml) for different periods of time. Methylene blue stain was performed to evaluate the resorption area on bone slices. Bleach solution was added to 96-well osteo surface plates to remove cells. Detailed analysis of pit formation area was described in our previous study^10^. The percentage of resorption area on osteo surface and bone slice was quantified using image J software (ver. 1.47).

### qPCR

Total RNA was isolated using Trizol reagent (Life Technologies). Single-stranded cDNA was prepared from 1 μg of total RNA using reverse transcriptase with oligo-dT primer according the manufacturer’s instructions (Promega, USA). Two microlitres of each cDNA was subjected to PCR amplification using specific primers for *CD47, mitf, RANK, Src, c-fos, NF-kappaB, MMP9, OSCAR, TRAF6, PU-1, Akt, Erk, p38, NFATc1, DC-STAMP, OC-STAMP, CD9, Ctsk, IRF8, BLIMP1* and *ATP6v0d2* with detailed information in Table S5.

### Microarray analysis

Total RNA was extracted from RAW264.7 cells induced with RANKL (100 ng/mL) and M-CSF (50 ng/mL) at different time points according to the study design. RNA quantity and quality were measured by NanoDrop ND-1000. RNA integrity was assessed by standard denaturing agarose gel electrophoresis. Arraystar Mouse LncRNA Microarray V3.0 was adopted for detection of lncRNA and mRNA expression, 35,923 LncRNAs and 24,881 mRNAs were detected. Arraystar Mouse circRNA Array and Agilent miRNA microarray were adopted for profiling the circRNAs and miRNAs expression. In total, 1,797 circRNAs and 1191 miRNAs were detected. All the microarray analysis was performed by KangChen Bio-tech (Shanghai, China). In brief, sample labeling and array hybridization were performed according to the Agilent One-Color Microarray-Based Gene Expression Analysis protocol (Agilent Technology) with minor modifications. Briefly, mRNA was purified from total RNA after removal of rRNA (mRNA-ONLY™ Eukaryotic mRNA Isolation Kit, Epicentre). Then, each sample was amplified and transcribed into fluorescent cRNA along the entire length of the transcripts without 3′ bias utilizing a random priming method (Arraystar Flash RNA Labeling Kit, Arraystar). The labeled cRNAs were purified by RNeasy Mini Kit (Qiagen). The concentration and specific activity of the labeled cRNAs (pmol Cy3/μg cRNA) were measured by NanoDrop ND-1000. 1 μg of each labeled cRNA was fragmented by adding 5 μl 10 × Blocking Agent and 1 μl of 25 × Fragmentation Buffer, then heated the mixture at 60 °C for 30 min, finally 25 μl 2 × GE Hybridization buffer was added to dilute the labeled cRNA. 50 μl of hybridization solution was dispensed into the gasket slide and assembled to the LncRNA expression microarray slide. The slides were incubated for 17 hours at 65 °C in an Agilent Hybridization Oven. The hybridized arrays were washed, fixed and scanned with using the Agilent DNA Microarray Scanner (part number G2505C). Agilent Feature Extraction software (version 10.7.3.1) was used to analyze acquired array images. Raw signal intensities were normalized in quantile method. Quantile normalization and subsequent data processing were performed using the GeneSpring GX v11.5.1 software package (Agilent Technologies).

### Differential lncRNA, mRNA, circRNA and miRNA screen and clustering analysis

GeneSpring software (V. 12.5) was adopted for normalization of the raw data from each array result. Differentially expressed lncRNA, mRNA, circRNA and miRNA were screened with p-value less than 0.05 and fold change more than 2.0. Difference integration analysis (Venn analysis) was then performed. The often characteristic elements between the four groups were determined by Venn analysis. Often up and down regulated RNAs were showed in pies with different colors. Differentially expressed lncRNAs, mRNAs, circRNAs and miRNAs were analyzed using Cluster software (V. 3.0). Normalized expression level of each RNA type was further analyzed with hierarchical clustering (HCL). The results were presented using Tree view software (V. 1.5).blue-white indicates lower expression, and red indicates high expression.

### GO & pathway analysis

DAVID (Database for Annotation, Visualization and Integrated Discovery) was used to analyze the potential functions of lncRNAs and co-expressed genes. The lncRNA function was predicted by GO functional annotation of co-expressed genes. Gene functions were classified into three subgroups namely biological process (BP), cellular component (CC) and molecular function (MF). GO terms with p-value less than 0.05 were selected and integrated using Venn analysis. The top 10 enriched often GO terms among the four groups ranked by fold enrichment and enrichment score were presented. KEGG pathway analysis was performed to determine the involvement of co-expressed genes in different biological pathways. Venn analysis was made to reveal the often pathways for up or down regulated co-expressed genes.

### lncRNA-mRNA and circRNA-miRNA co-expression network

The lncRNA-mRNA co-expression networks were built based on time dependent positive or negative correlations according to the normalized signal intensity of individual transcripts. The data were preprocessed by using the median gene expression intensity of all transcripts expressed from the same coding gene, without special treatment of the lncRNA expression value. Differentially expressed lncRNAs and mRNAs were then screened and removed from the dataset. Pearson’s correlation coefficient value was calculated for lncRNA-mRNA pairs, and strong correlated pairs (0.8 or greater) were included (either positive or negative) in the co-expression network^50^,^51^. A p-value of less than 0.05 was considered statistically significant. A total 241 lncRNAs and mRNAs containing 334 relationships were selected to generate a network map with cytoscape software (V. 3.2.1). Circle nodes represent lncRNAs and square nodes represent mRNAs. Red color and green color represent up and down regulation respectively. The shade darkness of red and green represent fold changes of lncRNAs. The size of circle represents p-value with larger size owing smaller p-value.

Similar with the lncRNA-mRNA network construction, the circRNA-miRNA co-expression network was constructed based on the correlation analysis between the differentially expressed circRNA and miRNAs. The expressions of differentially expressed circRNAs and miRNAs were analyzed by Pearson’s correlation coefficient. The absolute coefficient value of 0.8 between a circRNA and an miRNA was considered relevant for network construction. A p-value of less than 0.05 was considered statistically significant. A total 24 circRNAs and 82 miRNAs containing 95 relationships were selected to generate a network map. A diamondnode represents a circRNA and a circle node represents an miRNA. Yellow color and blue color represents up and down regulation respectively. The size of diamonds represents fold change of circRNAs with larger size owing higher fold change.

### Statistics

All data are representative of at least three experiments of similar results performed in triplicate unless otherwise indicated. Data are expressed as mean ± SD. One-way ANOVA followed by Student-Newman-Keuls post hoc tests was used to determine the significance of difference between results, with *p* < 0.05 being regarded as significant.

## Additional Information

**How to cite this article**: Dou, C. *et al.* Changing expression profiles of lncRNAs, mRNAs, circRNAs and miRNAs during osteoclastogenesis. *Sci. Rep.* **6**, 21499; doi: 10.1038/srep21499 (2016).

## Supplementary Material

Supplementary Information

Supplementary Dataset 1

Supplementary Dataset 2

Supplementary Dataset 3

Supplementary Dataset 4

Supplementary Dataset 5

Supplementary Dataset 6

Supplementary Dataset 7

Supplementary Dataset 8

Supplementary Dataset 9
