# Supplementary material for: Changing expression profiles of lncRNAs, mRNAs, circRNAs and miRNAs during osteoclastogenesis
Source: Sci Rep. 2016 Feb 9;6:21499. doi: 10.1038/srep21499 (PMC4746671; doi:10.1038/srep21499)
Supplement: Supplementary Information [file srep21499-s1.doc]

**Changing expression profiles of lncRNAs, mRNAs, circRNAs and miRNAs during osteoclastogenesis**

Ce Dou, ^1,2^ Zhen Cao,^2^ Bo Yang,^3^ Ning Ding, ^3^ Tianyong Hou, ^1^ Fei Luo, ^1^ Fei Kang,^2^ Jianmei Li, ^2^ Xiaochao Yang,^2^ Hong Jiang, ^2^ Junyu Xiang, ^2^ Hongyu Quan, ^2^ Jianzhong Xu,^1*^ AND Shiwu Dong^2*^

^1^National & Regional United Engineering Laboratory of Tissue Engineering, Department of Orthopedics, Southwest Hospital, Third Military Medical University, Chongqing, China.

^2^National & Regional United Engineering Laboratory of Tissue Engineering, Department of Biomedical Materials Science, School of Biomedical Engineering, Third Military Medical University, Chongqing, China.

^3^Department of Anatomy, Third Military Medical University, Chongqing, China.

**Supplementary Figures**


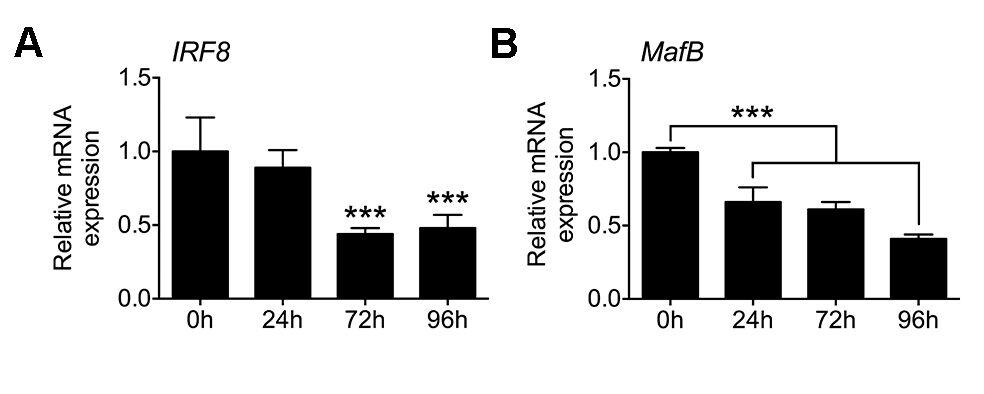


**Supplementary Fig. 1: qPCR results of *IRF8* and *MafB* for *in vitro* model validation.** To further confirm the in vitro model we established with RAW264.7 cells, two negatively regulated mRNAs during OCgenesis were detected. (**A**) Relative mRNA expression of *IRF8*normalized to GAPDH. (**B**) Relative mRNA expression of *MafB* normalized to GAPDH. The data in the figures represent the averages ± SD. **p*< 0.05, ***p*< 0.01, and ****p*< 0.001 based on one-way ANOVA.


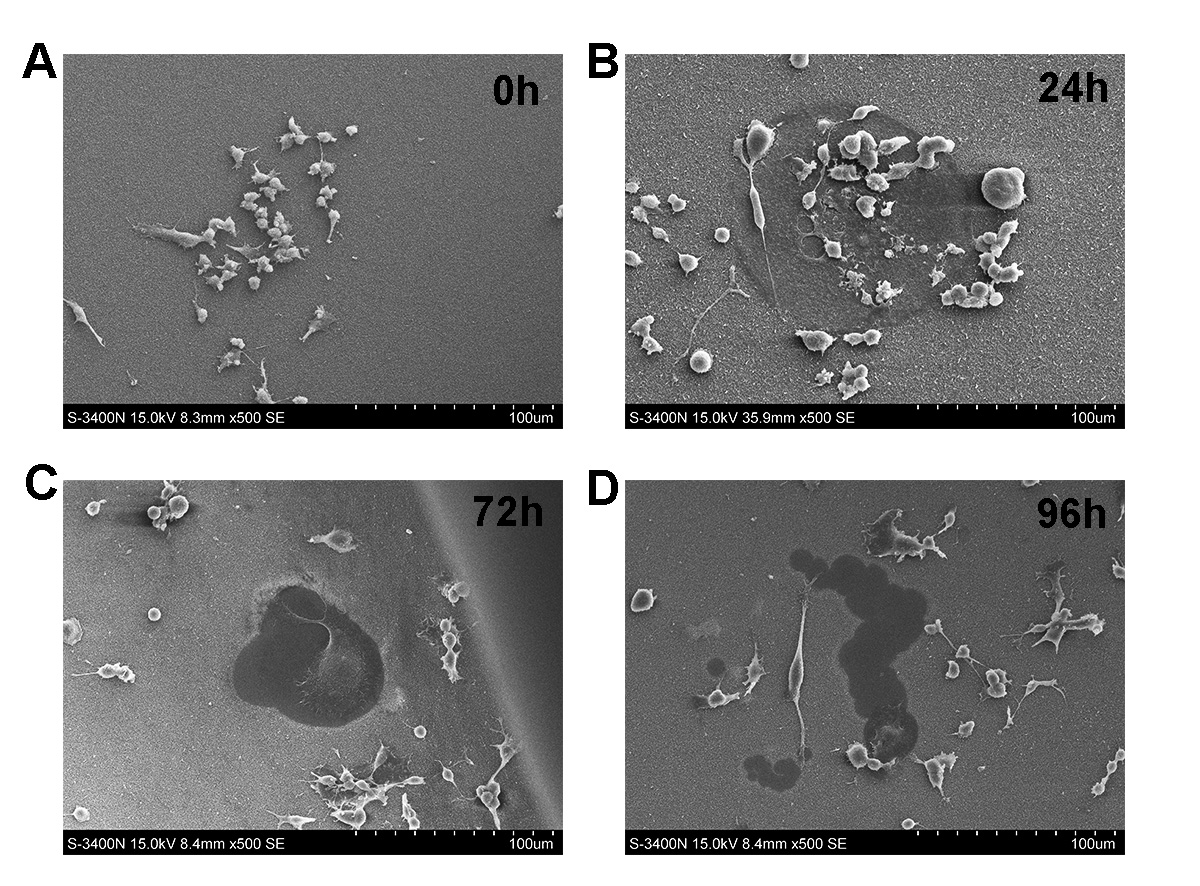


**Supplementary Fig. 2: Scanning Electron Microscope (SEM) analysis of pit formation assay.** RAW264.7 cells were induced with RANKL (100 ng/ml) and M-CSF (50 ng/ml) for 0, 24 h, 72 h and 96 h on bone surface slides. SEM was adopted for pit formation analysis. (**A**) RAW264.7 cells without RANKL and M-CSF induction. (**B**) Cells induced for 24 h on bone surface slides. (**C**) Cells induced for 72 h on bone surface slides. (**D**) Cells induced for 96 h on bone surface slides.


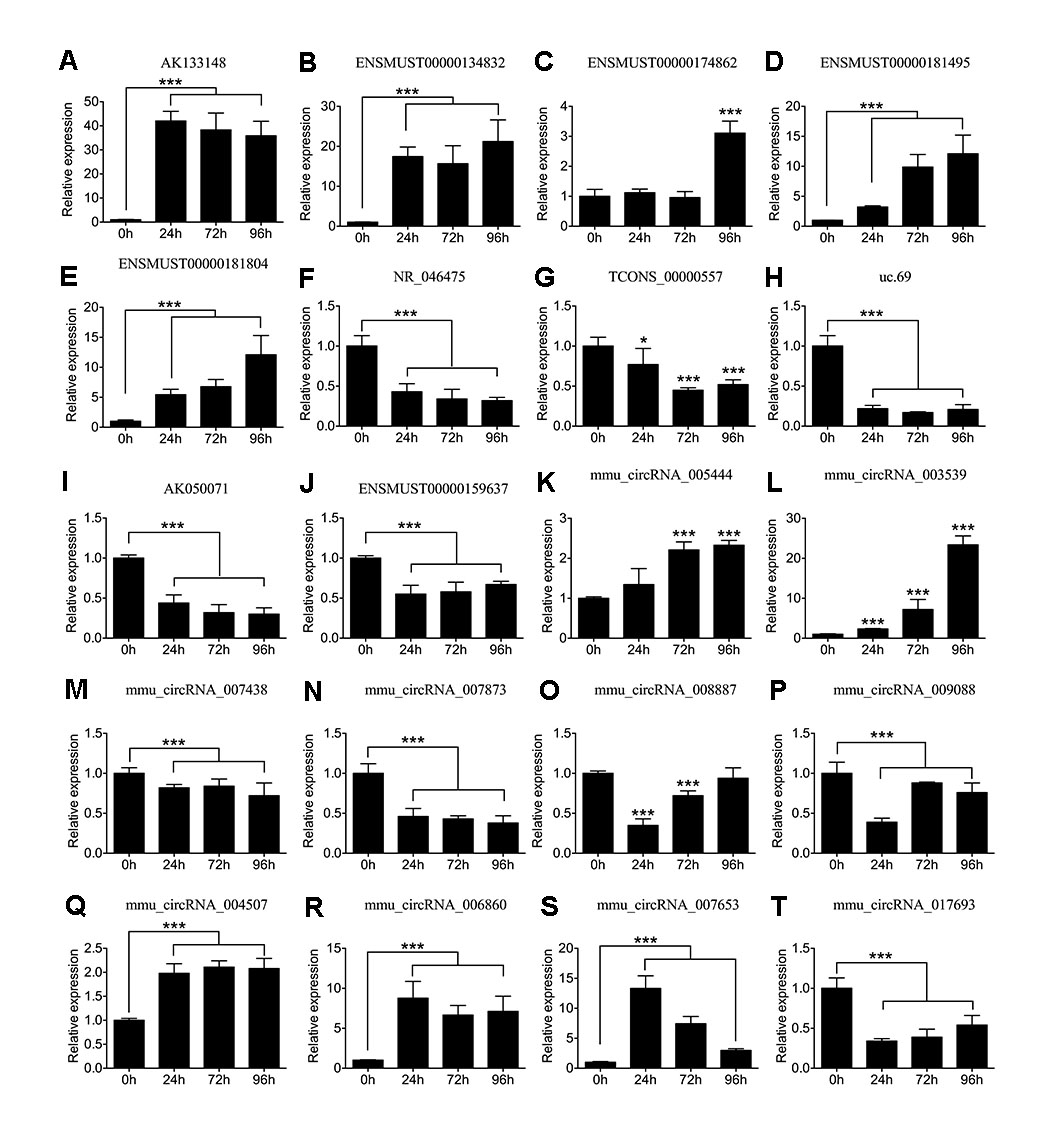


**Supplementary Fig. 3: Validation of the differences in lncRNAs and circRNAs.** To validate the differentially expressed lncRNAs and circRNAs suggested by the microarray results. qPCR was performed to test the expressions of 5 commonly up regulated lncRNAs, circRNAs and commonly down regulated lncRNAs and circRNAs. (**A-E**) Relative expression of the 5 commonly up regulated lncRNAs among all comparison groups (0 v24h, 0 v 72h, 0 v 96h) normalized to GAPDH. (**F-J**) Relative expression of the 5 commonly down regulated lncRNAs among all comparison groups normalized to GAPDH. (**K-O**) Relative expression of the 5 commonly up regulated circRNAs among all comparison groups normalized to GAPDH. (**P-T**) Relative expression of the 5 commonly down regulated circRNAs among all comparison groups normalized to GAPDH. The data in the figures represent the averages ± SD. **p*< 0.05, ***p*< 0.01, and ****p*< 0.001 based on one-way ANOVA.

**
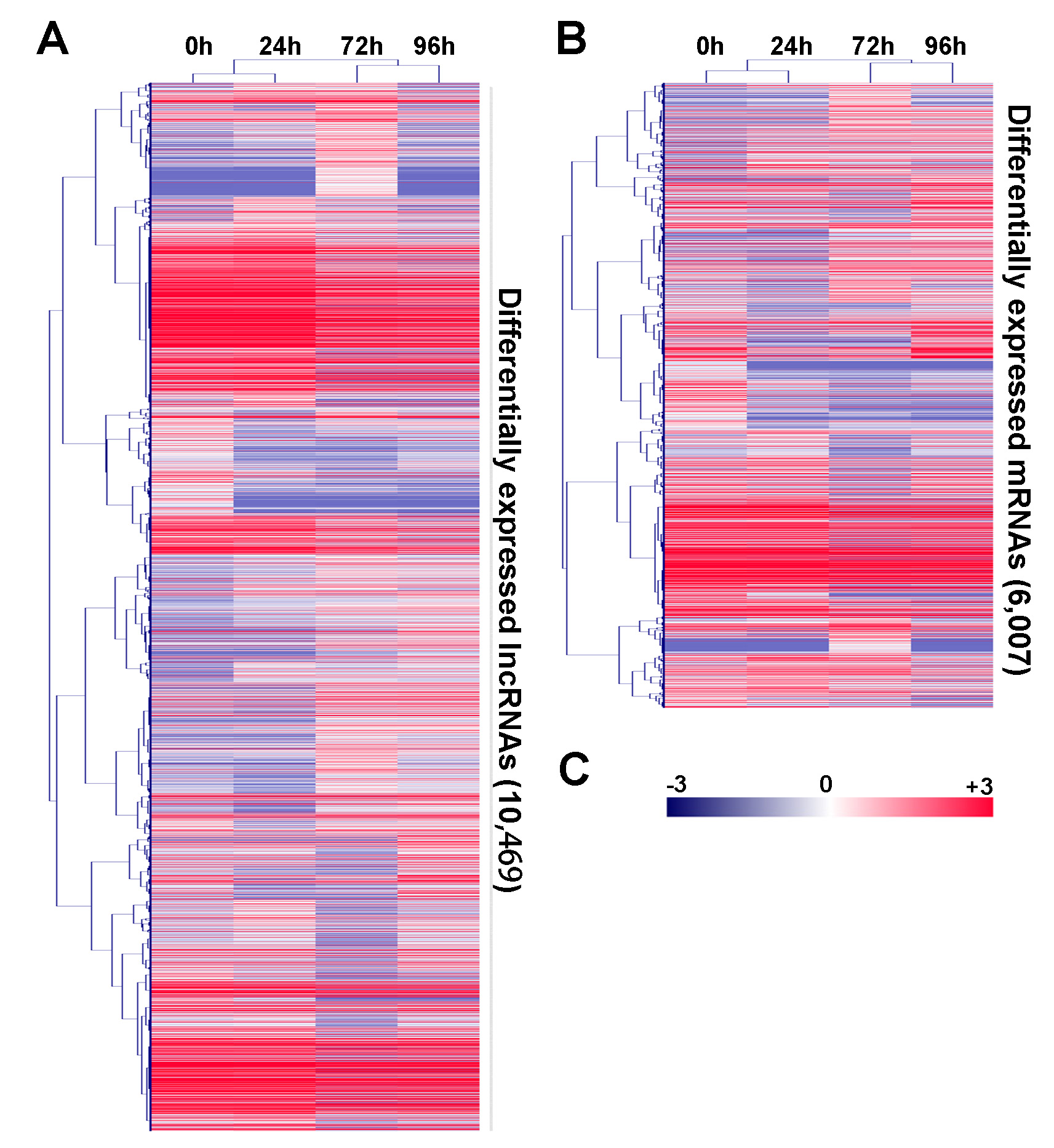
**

**Supplementary Fig. 4: Differentially expressed lncRNAs and mRNAs during osteoclasts differentiation and fusion. (A)** The cluster heat map of all differentially expressed lncRNAs expression at different stages during osteoclastogenesis from microarray data. **(B)** The cluster heat map of all differentially expressed mRNAs expression at different stages during osteoclastogenesis from microarray data. **(C)** Scale bar.

**
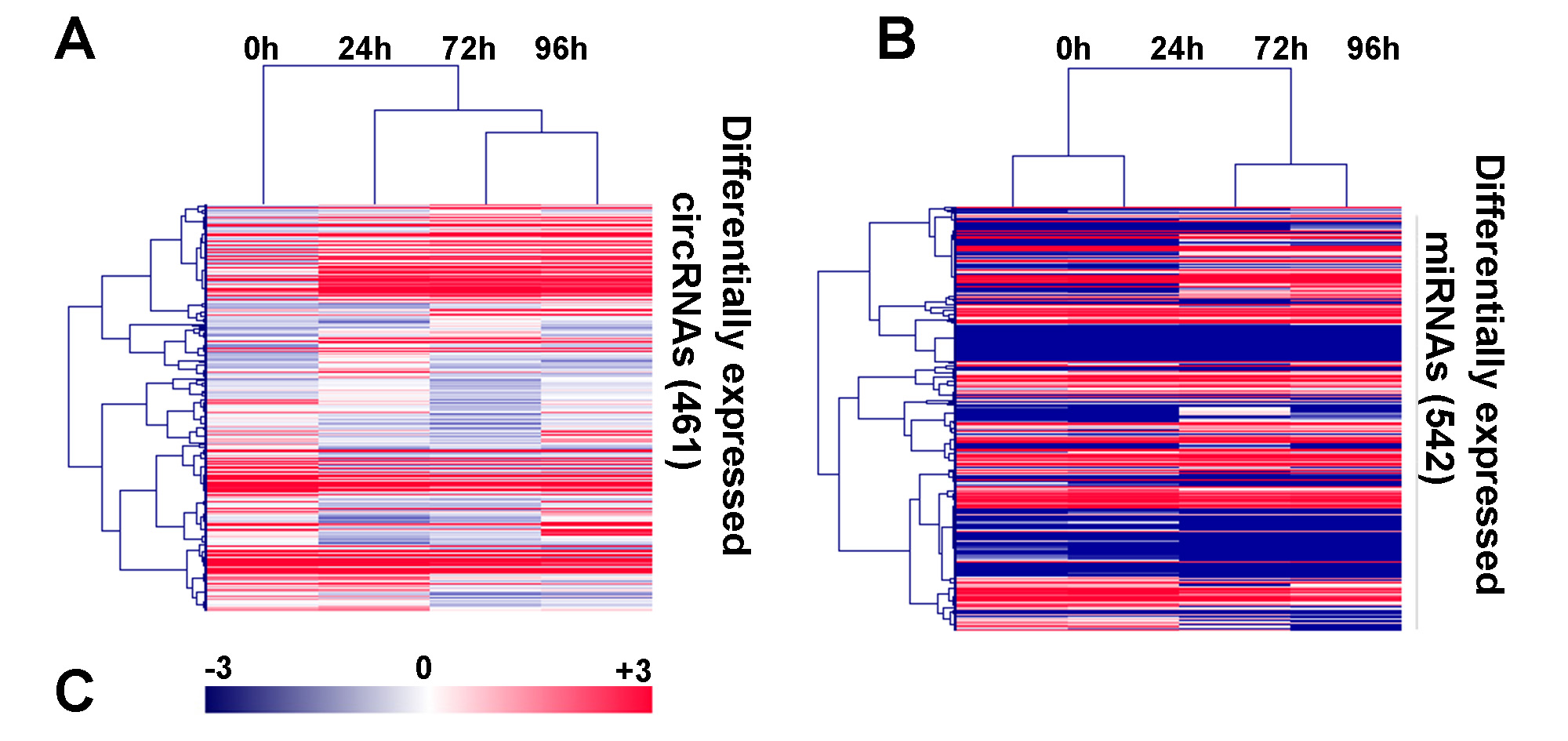
**

**Supplementary Fig. 5: Differentially expressed circRNAs and miRNAs during osteoclasts differentiation and fusion. (A)** The cluster heat map of all differentially expressed circRNAs expression at different stages during osteoclastogenesis from microarray data. **(B)** The cluster heat map of all differentially expressed miRNAs expression at different stages during osteoclastogenesis from microarray data. **(C)** Scale bar.

**
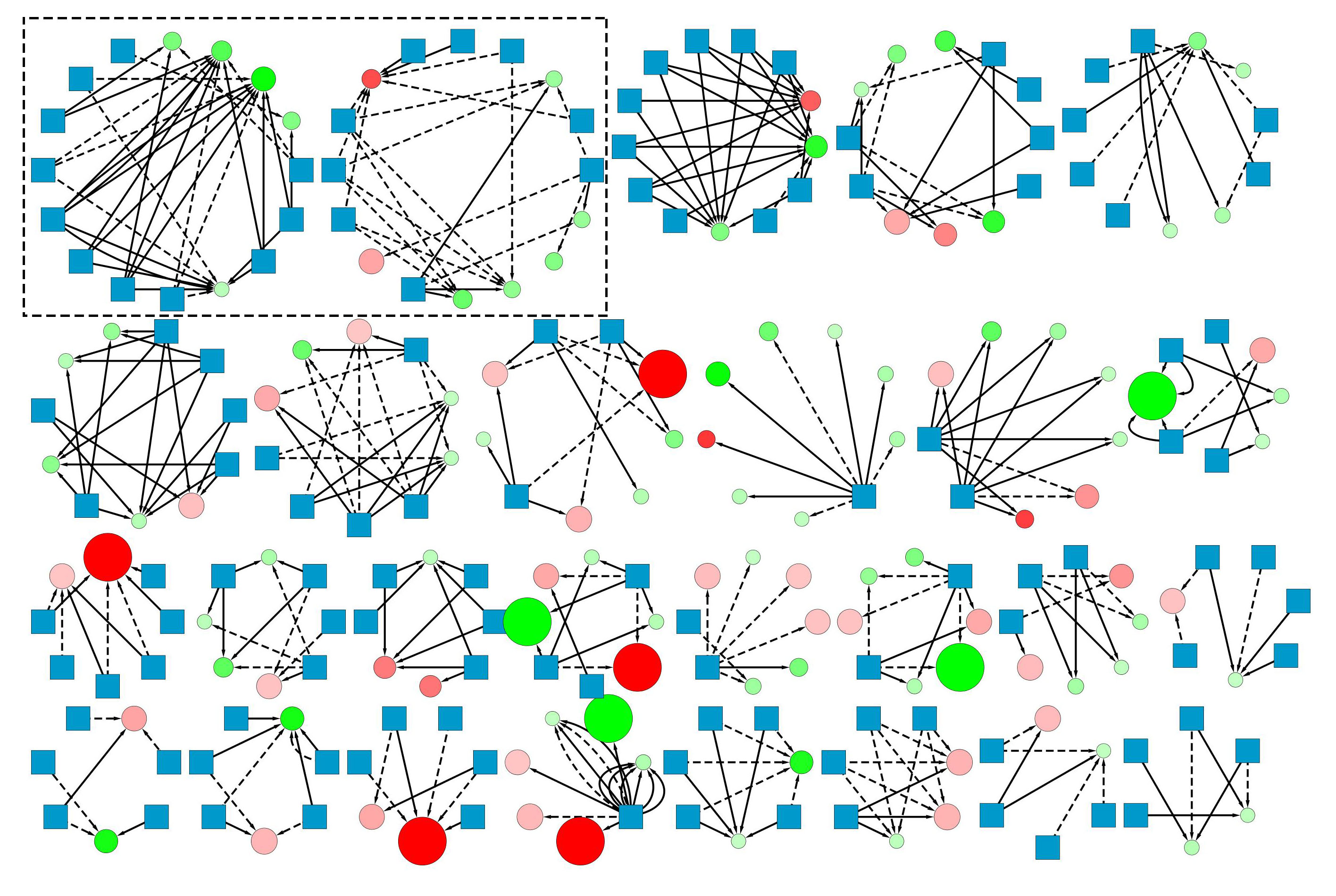
**

**Supplementary Fig. 6: Construction of the lncRNA-mRNA co-expression network.** Construction of the lncRNA-mRNA co-expression network. A circle node represents lncRNA and a square node represents mRNAs. Red color and green color represents up and down regulation respectively. The shade darkness of red and green represents fold change of lncRNAs. The size of circle represents p-value with larger size owing smaller p-value. Solid lines represent positive relationship and dash lines represent negative relationship.

**
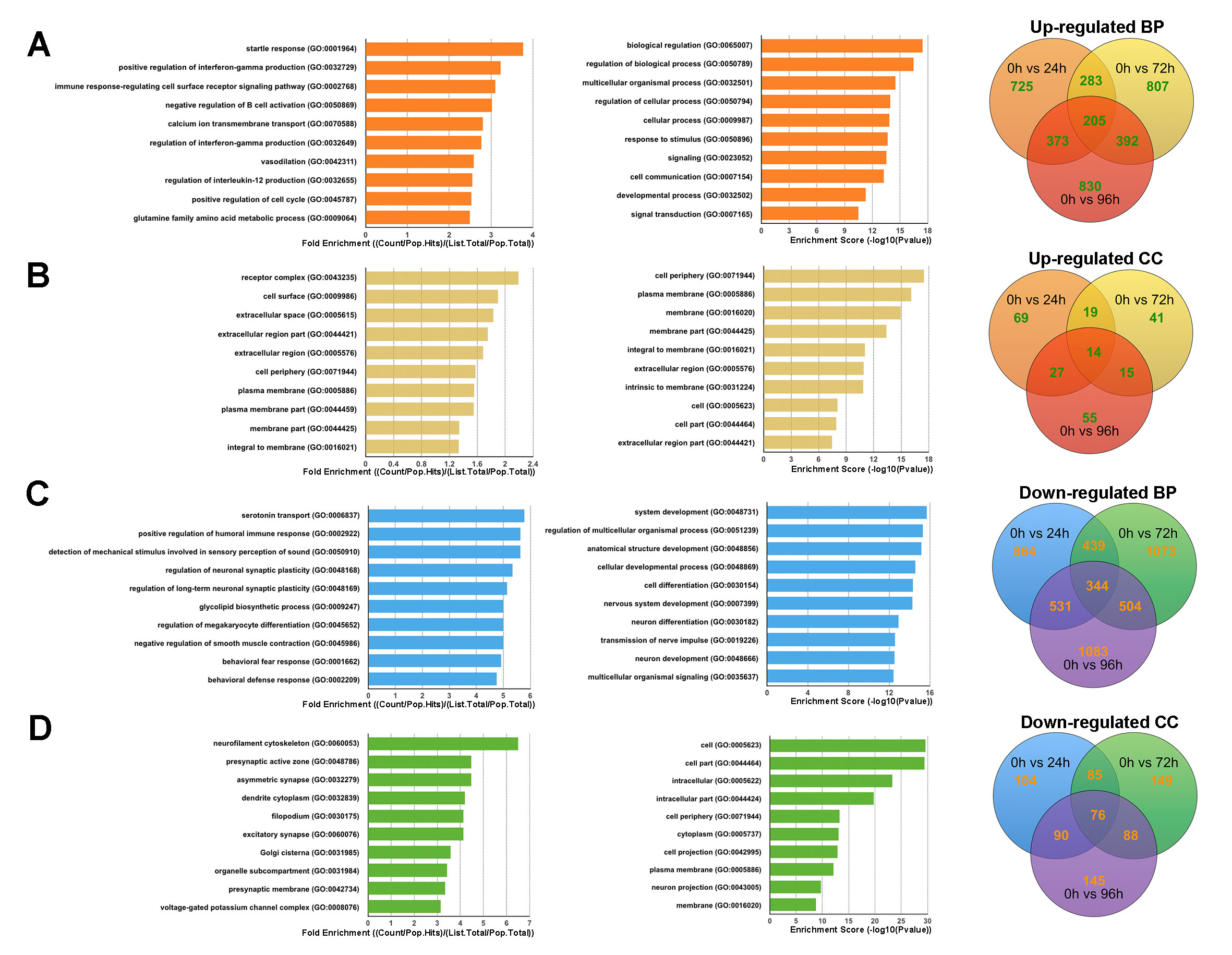
**

**Supplementary Fig. 7: GO analysis of the biological function of lncRNA co-expression genes.** (**A-B**) Common up regulated GO BP and CC terms for the difference lncRNAs co-expression genes were analyzed. Top 10 common up regulated GO terms ranked by fold enrichment and enrichment score were shown. (**C-D**) Common down regulated GO BP and CC terms for the difference lncRNAs co-expression genes were analyzed. Top 10 common down regulated GO terms ranked by fold enrichment and enrichment score were shown.

**Supplementary Tables:**

Table S1. lncRNAs primer sequences for qPCR.

| lncRNAs | Forward | Reverse | Tm(℃) |
| --- | --- | --- | --- |
| AK133148 | 5'-GACCCACCAGCCTACCCTAC-3' | 5'-AGCAGCACTTCAGGGACATT-3' | 60 |
| ENSMUST00000134832 | 5'-TGGTACTTCGTTGTCGCTGA-3' | 5'-ACCAGGCATGTAAGGATTGG-3' | 60 |
| ENSMUST00000181804 | 5'-GCCACAAGACACACAGCATT-3' | 5'-TCTTAACGCCAAAGCAGGAT-3' | 60 |
| ENSMUST00000174862 | 5'-CATCAGGAACTCAGCCAACA-3' | 5'-TTAGGGCCATGAAGCCAAT-3' | 60 |
| ENSMUST00000181495 | 5'-GCTCCTGAAGACGGAGACAC-3' | 5'-CCTGGCTTCCATTCTCTTGA-3' | 60 |
| uc.69- | 5'-TCCTGTTCGACAAAGCAACA-3' | 5'-CCGCGAATCTTCAGTCTCTC-3' | 61 |
| AK050071 | 5'-AGTGCTGATGCTGTGGTCTG-3' | 5'-TTTGTGTGTGCATGTGGTTG-3' | 60 |
| ENSMUST00000159637 | 5'-GCACTGAACAAGCCATTCAC-3' | 5'-GGGCCTGAGAAAGCTGAGTA-3' | 59 |
| NR_046475 | 5'-CCTGAAGAAAGAGCCACAGG-3' | 5'-GCCTCGGGAAGAAAGAGAAC-3' | 59 |
| TCONS_00000557 | 5'-GGTGTGGAGTGATTGGCTTT-3' | 5'-TTCCTAGACGACACGCTCAA-3' | 59 |

Table S2. circRNAs primer sequences for qPCR.

| circRNAs | Forward | Reverse | Tm(℃) |
| --- | --- | --- | --- |
| mmu_circRNA_007438 | 5'-CGGTGTCTTTCTCCACAT-3' | 5'-CTATATACGCCATGGAAATTA-3' | 53 |
| mmu_circRNA_007873 | 5'-CTCGGAGTTTGCTTCATC-3' | 5'-CAATGAGAACCAGTACCCA-3' | 55 |
| mmu_circRNA_008887 | 5'-CAGTGGATCTCTTCTGGAA-3' | 5'-CGATGTGTGCACGAGGA-3' | 54 |
| mmu_circRNA_009088 | 5'-CTCCTTAATTTGCAATTGCC-3' | 5'-AGGAGACAAAAAGAAAGACA-3' | 52 |
| mmu_circRNA_017693 | 5'-TAAGGGCTGGAATAAAACAT-3' | 5'-TGCAATGGTCTCTGCAAG-3' | 53 |
| mmu_circRNA_015622 | 5'-TCTTTTCTAACCGTTTTCTG-3' | 5'-ATGCAGACACCAAAAGGC-3' | 53 |
| mmu_circRNA_016157 | 5'-CTCAGTAAGTTCTGGCTC-3' | 5'-CTATCAGACAGTGAGGTG-3' | 54 |
| mmu_circRNA_005108 | 5'-TCCCTCTCCTTCCTCCCT-3' | 5'-GACTTGAACCCCGCGAG-3' | 57 |
| mmu_circRNA_006518 | 5'-TTTAAATTTCCTGGCACGCA-3' | 5'-AGCGGTGGGCCTGGC-3' | 55 |
| mmu_circRNA_010763 | 5'-CCCAGTGTTAAGTGCTTTC-3' | 5'-GGTCAGCAAATGCCCCT-3' | 55 |

Table S3. Up regulated KEGG pathway analysis

| **PathwayID** | | **Definition** | **Counts** | | **Enrichment_Score** | **P-value** |
| --- | --- | --- | --- | --- | --- | --- |
| mmu04060 | Cytokine-cytokine receptor interaction - Mus musculus (mouse) | | | 29 | 1.952653 | 0.01115184 |
| mmu04015 | Rap1 signaling pathway - Mus musculus (mouse) | | | 26 | 2.474987 | 0.003349757 |
| mmu05202 | Transcriptional misregulation in cancer - Mus musculus (mouse) | | | 23 | 6.049636 | 8.91999E-07 |
| mmu04151 | PI3K-Akt signaling pathway - Mus musculus (mouse) | | | 23 | 1.767568 | 0.0170778 |
| mmu04380 | Osteoclast differentiation - Mus musculus (mouse) | | | 22 | 4.415815 | 3.83871E-05 |
| mmu04080 | Neuroactive ligand-receptor interaction - Mus musculus (mouse) | | | 21 | 1.784172 | 0.01643719 |
| mmu04142 | Lysosome - Mus musculus (mouse) | | | 17 | 2.383796 | 0.004132417 |
| mmu04310 | Wnt signaling pathway - Mus musculus (mouse) | | | 17 | 1.747267 | 0.01789505 |
| mmu04514 | Cell adhesion molecules (CAMs) - Mus musculus (mouse) | | | 17 | 1.333592 | 0.04638823 |
| mmu04015 | Rap1 signaling pathway - Mus musculus (mouse) | | | 15 | 1.491935 | 0.03221554 |
| mmu04064 | NF-kappa B signaling pathway - Mus musculus (mouse) | | | 14 | 4.279204 | 5.2577E-05 |
| mmu04620 | Toll-like receptor signaling pathway - Mus musculus (mouse) | | | 13 | 3.636082 | 0.000231163 |
| mmu04668 | TNF signaling pathway - Mus musculus (mouse) | | | 13 | 3.307034 | 0.000493135 |
| mmu05323 | Rheumatoid arthritis - Mus musculus (mouse) | | | 12 | 2.021115 | 0.009525448 |
| mmu04630 | Jak-STAT signaling pathway - Mus musculus (mouse) | | | 11 | 1.396778 | 0.04010721 |
| mmu04660 | T cell receptor signaling pathway - Mus musculus (mouse) | | | 9 | 1.54911 | 0.02824166 |
| mmu04066 | HIF-1 signaling pathway - Mus musculus (mouse) | | | 9 | 1.414995 | 0.0384596 |
| mmu04150 | mTOR signaling pathway - Mus musculus (mouse) | | | 8 | 2.485581 | 0.003269031 |
| mmu04666 | Fc gamma R-mediated phagocytosis - Mus musculus (mouse) | | | 8 | 1.558729 | 0.02762301 |

Table S4.Down regulated KEGG pathway analysis

| PathwayID | Definition | Counts | Enrichment_Score | P-value |
| --- | --- | --- | --- | --- |
| mmu04080 | Neuroactive ligand-receptor interaction - Mus musculus (mouse) | 50 | 5.081911 | 8.28111E-06 |
| mmu04060 | Cytokine-cytokine receptor interaction - Mus musculus (mouse) | 34 | 2.09698 | 0.007998714 |
| mmu04010 | MAPK signaling pathway - Mus musculus (mouse) | 29 | 1.411063 | 0.0388094 |
| mmu04020 | Calcium signaling pathway - Mus musculus (mouse) | 28 | 3.107261 | 0.000781157 |
| mmu04514 | Cell adhesion molecules (CAMs) - Mus musculus (mouse) | 22 | 1.973744 | 0.01062322 |
| mmu04015 | Rap1 signaling pathway - Mus musculus (mouse) | 22 | 1.598339 | 0.02521511 |
| mmu04540 | Gap junction - Mus musculus (mouse) | 18 | 3.725738 | 0.000188045 |
| mmu04066 | HIF-1 signaling pathway - Mus musculus (mouse) | 15 | 1.444064 | 0.03596966 |
| mmu04512 | ECM-receptor interaction - Mus musculus (mouse) | 14 | 1.9813 | 0.01043998 |
| mmu04971 | Gastric acid secretion - Mus musculus (mouse) | 14 | 3.58031 | 0.000262839 |
| mmu04915 | Estrogen signaling pathway - Mus musculus (mouse) | 14 | 2.352907 | 0.004437033 |
| mmu04610 | Complement and coagulation cascades - Mus musculus (mouse) | 12 | 1.666193 | 0.02156785 |
| mmu04622 | RIG-I-like receptor signaling pathway - Mus musculus (mouse) | 11 | 2.365827 | 0.004306985 |
| mmu04912 | GnRH signaling pathway - Mus musculus (mouse) | 11 | 1.52304 | 0.02998886 |
| mmu04070 | Phosphatidylinositol signaling system - Mus musculus (mouse) | 10 | 1.423125 | 0.03774637 |
| mmu04612 | Antigen processing and presentation - Mus musculus (mouse) | 10 | 1.423125 | 0.03774637 |
| mmu04623 | Cytosolic DNA-sensing pathway - Mus musculus (mouse) | 9 | 1.686917 | 0.02056285 |
| mmu04975 | Fat digestion and absorption - Mus musculus (mouse) | 8 | 1.850938 | 0.0140949 |
| mmu04961 | Endocrine and other factor-regulated calcium reabsorption - Mus musculus (mouse) | 8 | 1.592162 | 0.02557629 |

Table S5. Primer sequences for PCR

| **Genes** | **Forward** | **Reverse** | **Tm (°C)** |
| --- | --- | --- | --- |
| DC-STAMP | 5'-TTATGTGTTTCCACGAAGCCCTA-3' | 5'-ACAGAAGAGAGCAGGGCAACG-3' | 62 |
| CD9 | 5'-CGGTCAAAGGAGGTAG-3' | 5'-GGAGCCATAGTCCAATA-3' | 60 |
| ATP6v0d2 | 5'-AGCAAAGAAGACAGGGAG-3' | 5'-CAGCGTCAAACAAAGG-3' | 60 |
| OC-STAMP | 5'-GGGCTACTGGCATTGCTCTTAGT-3' | 5'-CCAGAACCTTATATGAGGCGTCA-3' | 62 |
| CD47 | 5'-TGGTGGGAAACTACACTTGCGA-3' | 5'-AGGCTGATCCTTGGTCAGTGTTG-3' | 63 |
| mitf | 5'-GTGCAGACCCACCTGGAAAAC-3' | 5'-AGTTAGAGTGAGCATAGCCTAG-3' | 61 |
| RANK | 5'-TACCACCTGACAACAGTACC-3' | 5'-ATCCATCAGAAATACAGCAC-3' | 64 |
| Nfkb | 5'-AGCCTCAGGGCCTTACAGA-3' | 5'-CCGTACAATGCTCAGATCCA-3' | 61 |
| MMP9 | 5'-ACCCGAAGCGGACATT-3' | 5'-GGCATCTCCCTGAACG-3' | 62 |
| Ctsk | 5'-GCGGCATTACCAACAT-3' | 5'-CTGGAAGCACCAACGA-3' | 61 |
| Akt | 5'-TCGTGTGGCAGGATGTGTAT-3' | 5'-ACCTGGTGTCAGTCTCAGAGG-3' | 62 |
| c-fos | 5‘-AGGCAGAACCCTTTGA-3’ | 5’-GGTGACCACGGGAGTA-3‘ | 60 |
| p38 | 5'-GTGCCCGAGCGTTACCAGACC-3' | 5'-CTGTAAGCTTCTGACATTTC-3' | 62 |
| Erk | 5'-AATCCCCGTTGGTCTTACAC-3' | 5'-CCTAAGGAAAAG-CTCAAAGA-3' | 61 |
| NFATc1 | 5'-GAGGAGTTGGCTCAGTG-3' | 5'-TAGCGTTCCGTTCGTT-3' | 61 |
| OSCAR | 5'-GGTCCTCATCTGCTTG-3' | 5'-TATCTGGTGGAGTCTGG-3' | 62 |
| PU.1 | 5'-CACGTCCTCGATACTCCCATG-3' | 5'-CTCTGCCTCTCACCCTCCTCC-3' | 61 |
| src | 5'-TTTGGCAAGATCACTAGACGGG-3' | 5'-GAGGCAGTAGGCACCTTTTGT-3' | 59 |
| TRAF6 | 5'-TTGCACATTCAGTGTTTTTGG-3' | 5'-TGCAAGTGTCGTGCCAAG-3' | 60 |
